# Four new additions to *Helvella* (Helvellaceae, Pezizales) from Northern Thailand

**DOI:** 10.3389/fmicb.2023.1182025

**Published:** 2023-07-03

**Authors:** Feng-Ming Yu, Lei Lei, Thatsanee Luangharn, Qi Zhao, Ying-An Zhu

**Affiliations:** ^1^College of Landscape and Horticulture, Yunnan Agricultural University, Kunming, Yunnan, China; ^2^Key Laboratory for Plant Diversity and Biogeography of East Asia, Kunming Institute of Botany, Chinese Academy of Sciences, Kunming, Yunnan, China; ^3^Center of Excellence in Fungal Research, Mae Fah Luang University, Chiang Rai, Thailand; ^4^School of Science, Mae Fah Luang University, Chiang Rai, Thailand

**Keywords:** new taxa, phylogeny, saddle fungi, species diversity, systematics

## Abstract

Most species of *Helvella* have been described from temperate regions in Asia, Europe, and North America, but little is known about the genus from tropical regions. In this report, phylogenetic analyses of 11 newly collected saddle-like fungi from northern Thailand using three genetic markers [the nuclear large subunit ribosomal DNA (LSU), the heat shock protein 90 (*HSP*90), and the translation elongation factor 1-alpha (*TEF*)] confirm their assignment in *Helvella*. Two species were described as new, i.e., *Helvella atroides* and *H. orentitomentosa*, and two species, i.e., *H*. *fistulosa* and *H. rugosa*, were reported for the first time in Thailand. Details of macro- and microscopic characters and illustrations were provided for each species. To date, seven species of *Helvella* have been recorded in Thailand, and a key for identifying the Thai *Helvella* species was provided here.

## 1. Introduction

Helvellaceae Fr. (Pezizales, Pezizomycetes, Ascomycota) was established by Fries (1822) and encompasses taxa that produce some epigeous apothecial forms and hypogeous ascomata. Currently, Helvellaceae comprises six genera, i.e., *Balsamia* Vittad. (syn. *Barssia* Gilkey), *Dissingia* K. Hansen, X.H. Wang & T. Schumach., *Helvella* L., *Pindara* Velen., *Midotis* Fr. (syn. *Wynnella* Boud.), and *Underwoodia* Peck *sensu stricto* (Hansen et al., 2019).

*Helvella* L., the elfin saddle mushroom, is the largest genus in the family Helvellaceae and is typified by *Helvella crispa* (Scop.) Fr. (Linnaeus, 1753; Fries, 1822; Hansen et al., 2019). *Helvella* is distributed worldwide, and members are mainly found in mountainous and forested regions in north-temperate Eurasia and North America (Zhao et al., 2015, 2016a; Skrede et al., 2017). The genus includes a series of elaborate ascomata, from cupulate to saddle-shaped and/or from lobed to folded apothecia, which are located on terete, ribbed, or furrowed stipes (Skrede et al., 2017). *Helvella* ascomata usually produce a wide range of colors such as white, creamy white, gray, and brown to black (Landeros et al., 2012; Skrede et al., 2017). Some *Helvella* species form ectomycorrhizal symbioses with plants in several families, such as *Fagaceae, Pinaceae*, and *Salicaceae* (Nguyen et al., 2013; Hwang et al., 2015). In addition, *Helvella* is of economic value because some members have high edible properties (Dai et al., 2009; Ariyawansa et al., 2015; Zhao et al., 2015, 2016a). Two *Helvella*, namely *H*. *crispa* and *H*. *lacunosa* Afzel., are widely consumed as edible species (Dai et al., 2009), and *H*. *bachu* Q. Zhao, Zhu L. Yang & K.D. Hyde is preferred as the most notable edible species (Zhao et al., 2016a).

*Helvella sensu lato* was divided into six to eight infrageneric classifications, which were supported by morphology evidence (Dissing, 1966; Korf, 1972; Weber, 1972; Harmaja, 1979; Häffner, 1987; Abbott and Currah, 1997), and later, it was proved to be polyphyletic by molecular-based methods (Hansen et al., 2019). Based on the molecular analyses of a combination of LSU, *RPB*2, and *TEF* genes and a wide representative sample, Hansen et al. (2019) reinstated *Pindara* as a distinct genus and established a new genus *Dissingia* to accommodate sect. *Leucomelaena* lineage (previously placed in *Helvella s.l*.). In Index Fungorum (http://www.indexfungorum.org/names/Names.asp, accessed on 4 May 2023) and Species Fungorum (https://www.speciesfungorum.org/Names/names.asp, accessed on 4 May 2023), there are approximately 550 and 140 *Helvella* records, respectively. They represent taxa originally described as saddle fungi and later referred to or excluded from *Helvella* due to the numerous evidence of taxonomy, phylogeny, or nomenclature (Skrede et al., 2017). Recently, *Helvella s.s*. was updated by some authors and approximately 100 species were widely accepted (Skrede et al., 2017, 2020, 2023; Zhuang et al., 2018; Løken et al., 2019; Wang et al., 2019; Landeros et al., 2021; Xu et al., 2022).

In Europe, Skrede et al. (2017, 2020, 2023) and Løken et al. (2019) conducted a thorough investigation of saddle fungi and found at least 72 *Helvella* species are there. In Asia, research on the species diversity of *Helvella* is mainly concentrated in China, with more than 60 species recorded, mostly from southwest China (Ariyawansa et al., 2015; Zhao et al., 2015, 2016a,b; Hyde et al., 2016, 2020; Wang et al., 2016; Tibpromma et al., 2017; Zhuang et al., 2018; Xu et al., 2022). However, the attention paid to saddle fungi in tropical areas is limited, especially in Thailand. Boonthavikoon (1998) and Tibpromma et al. (2017) reported that three species were recorded in Thailand, i.e., *H. crispa, H. crispoides* Q. Zhao & K.D. Hyde, and *H. elastica* Bull. Given the high level of species diversity and provincialism discovered within saddle fungi and the rich fungal diversity in tropical regions, we hypothesize that there will be novel species lineages in these places.

In this study, we reported new knowledge of *Helvella* species collected from northern Thailand. The 11 *Helvella* collections are morphologically and phylogenetically analyzed here. The results of four species of *Helvella* were found, of which two were new species and the other two were recorded for the first time. A taxonomic key to *Helvella* taxa in Thailand was provided.

## 2. Materials and methods

### 2.1. Specimen and morphological studies

Collections were obtained in Chiang Mai and Chiang Rai Provinces, Thailand and photographed *in situ*. Microscopic observations and photomicrographs were made. The hemiamyloid reaction in Melzer's reagent is as follows: “J^+^” for a hemiamyloid (“solely red”) reaction and “J^−^” for a negative reaction. For microscopic examination, dried specimens were sliced manually and then rehydrated in water. A Nikon ECLIPSE 80i microscope was used for observation and microphotography. The notations “ascospores (n/m/p)” indicate that the measurements were made on “**n**” ascospores from “**m**” ascomata of “**p**” collections. The measurements of ascospores were indicated in (a–) b–c (–d), where the range b–c represents the 95% confidence interval, and a and b represent the minimum and maximum, respectively. Q refers to the length/breadth ratio of ascospores, and bold **Q** referred to the average Q of ascospores ± sample standard deviation. Examined specimens were deposited at Mae Fah Luang University, Chiang Rai, Thailand (MFLU). Index Fungorum numbers and Facesoffungi numbers were obtained as detailed in the Index Fungorum (http://www.indexfungorum.org/names/names.asp) and Jayasiri et al. (2015).

### 2.2. DNA extraction, PCR amplification, and sequencing

Genomic DNA was extracted from dried apothecia using the CTAB procedure with some modification (Doyle and Doyle, 1990). The large subunit of the nuclear ribosomal RNA (Partial LSU), the translation elongation factor 1-alpha (*TEF*), and the heat shock protein 90 (*HSP*90) were amplified by polymerase chain reaction (PCR) using universal and/or previously published primers LR0R/LR5 (Vilgalys and Hester, 1990) and H_LSUf1/H_LSUr2 (Landeros et al., 2015), EF595F/EF1160R (Skrede et al., 2017), and H_*hsp*f and H_*hsp*r (Skrede et al., 2017) (Table 1). PCR amplifications were performed in a total volume of 25 μl, containing 21 μl 1.1 × T3 Super PCR Mix (Tsingke TSE030, Tsingke Biological Technology Co.), 1 μl of each primer, and 2 μl of DNA template. PCR reactions were carried out in an Applied Biosystems 2720 Thermal Cycler (Foster City, CA, USA) under the following conditions: an initial denaturation at 98°C for 5 min, followed by 34 cycles of denaturation at 98°C for 25 s (30 s for LSU and *HSP*90), annealing at 53°C for 30 s (52°C for LSU: H_LSUf1/H_LSUr2, 58°C for *HSP*90), and extension 45 s at 72°C, followed by a final extension at 72°C for 7 min. PCR products were verified by electrophoresis with 1% ethidium bromide-stained agarose gel. Those presenting the target genes have been sent to Sangon Biotech (Shanghai) Co., Ltd., Shanghai, China, for sequencing.

**Table 1:** Genes and their corresponding primers used in this study.

**Locus**	**Forward primer sequence (5^′^-3^′^)**	**Reverse primer sequence (5^′^-3^′^)**	**References**
LSU	LR0R: ACCCGCTGAACTTAAGC	LR5: TCCTGAGGGAAACTTCG	Vilgalys and Hester, 1990
	H_LSUf1: AGCGGAGGAAAGAAACCAACA	H_LSUr2: TCCCAACAGCTATGCTCCTAC	Landeros et al., 2015
*TEF*	EF595F: CGTGACTTCATCAAGAACATG	EF1160R: CCGATCTTGTAGACGTCCTG	Skrede et al., 2017
*HSP*90	H_*hsp*f: CRGGCATCCGGGTGACGTAAT	H_*hsp*r: AGGGKGTTGTCGACTCCGAGG	Skrede et al., 2017

### 2.3. DNA sequence data analyses

The phylogenetic trees were constructed using the sequencing data of newly collected *Helvella* samples and the allied reference sequences of closely related saddle species obtained from the GenBank (Table 2). *Dissingia confusa* (O-253269, O-253268, KH.12.75) and *D*. *leucomelaena* (DMS-9190862, KH.06.01) were used as outgroup taxa. All sequences were assembled and aligned using MAFFT v. 7 (Kuraku et al., 2013; Katoh et al., 2019) and manually edited where necessary using BioEdit version 7.0.9 (Hall, 1999). Individual alignments were compiled for LSU, *HSP*90, and *TEF* genes. The optimal substitution model for each gene dataset was determined using MrModeltest 2.3 (Nylander, 2004) under the Akaike information criterion (AIC). The results indicated that the GTR+I+G model was optimal for LSU, SYM+G for *TEF*, and HKY+G for *HSP*90. Individual datasets were combined to assemble the combined dataset (gene order: LSU, *HSP*90, and *TEF*).

**Table 2:** Species names, voucher numbers, and corresponding GenBank accession numbers used in this study.

**Fungal species**	**Type**	**Sample ID/voucher**	**GenBank accession no**.		
			**LSU**	***HSP*** **90**	***TEF***
*Dissingia confusa*		O-253268	MK100254	KY784529	MK113873
*D. confusa*		KH.12.75	MK100255	/	MK113890
*D. confusa*		O-253269	MK100253	/	MK113872
*D. leucomelaena*		KH.06.01	KC012682	/	KC109207
*D. leucomelaena*		DMS-9190862	MK100257	/	MK113835
*Helvella atra*	Epitype	C Fungi Exs. Suec. 2066 (H406)	/	KY784502	/
*H. atra*		O-253251 (H016)	KY772911	KY784193	KY772828
*H. atra*		O-253245 (H233)	KY773063	KY784351	
*H. atra*		O-255762 (H1055)	MN655852	MN692348	MN689304
*H. atroides*	Holotype	MFLU23-0102	OQ866059	OQ863541	OQ863552
*H. atroides*		MFLU23-0103	OQ866060	OQ863542	OQ863553
*H. atroides*		MFLU23-0101	OQ866058	OQ863540	OQ863551
*H. bachu*	Holotype	HKAS 88105	NG_059662		KU739842
*H. convexa*	Holotype	H761 (UPS-F-145677)	OQ626670	OQ633415	/
*H. convexa*		H746 (UPS-F-145717)	OQ626669	OQ633414	/
*H. danica*	Holotype	C-F-85205 (H263)	KY773083	KY784378	/
*H. danica*		O-129543 (H177)	KY773022	KY784306	/
*H. danica*		O-253288 (H058)	KY772942	KY784222	/
*H. ephippioides*		O-253267 (H085)	KY772957	KY784237	KY772867
*H. fibrosa*		C-F-53774 (H357)	KY773125	KY784458	/
*H. fibrosa*		O-291352 (H240)	KY773069	KY784358	KY772898
*H. fibrosa*	Isoepitype	C (H413)	/	KY784508	/
*H. fistulosa*	Neotype	O-291887 (H241)	KY773070	KY784359	/
*H. fistulosa*		FH (H109)	KY772966	KY784248	/
*H. fistulosa*		O-253314 (H205)	KY773039	KY784329	/
*H. fistulosa*		MFLU23-0098	OQ866055	OQ863537	OQ863550
*H. fistulosa*		MFLU23-0097	OQ866054	OQ863536	/
*H. fistulosa*		MFLU23-0096	OQ866053	OQ863535	/
*H. hispanica*	Holotype	O-F-256537 (H1929)	/	MN598129	/
*H. hispanica*		O-F-256536 (H1023)	MN644504	MN598112	/
*H. japonica*	Holotype	H093 (O-F-253389)	KY772961	KY784243	/
*H. japonica*		H995 (S-F-126523)	OQ626667	OQ633413	/
*H. macropus*	Epitype	C Fungi Exs. Suec. 3266 (H412)	/	KY784507	/
*H. macropus*		O-253326 (H073)	KY772954	KY784233	KY772863
*H. macropus*		FH (H119)	KY772973	KY784255	KY772871
*H. macropus*		O-291425 (H238)	KY773067	KY784356	KY772896
*H. macropus*		O-291391 (H239)	KY773068	KY784357	KY772897
*H. monachella*	Epitype	C-F-92121 (H268)	/	KY784383	/
*H. monachella*		C-F-92120 (H269)	KY773084	KY784384	/
*H. neopallescens*	Holotype	O-F-256550 (H1022)	MN644500	MN598111	/
*H. neopallescens*		O-F-256551 (H1025)	/	MN598113	/
*H. neopallescens*		TRH-12607 (H2884)	MN644501	MN598174	/
*H. orentitomentosa*	Holotype	MFLU23-0099	OQ866056	OQ863538	/
*H. orentitomentosa*		MFLU23-0100	OQ866057	OQ863539	/
*H. pallescens*	Epitype	O-66205 (H138)	KY772988	KY784271	KY772878
*H. pallescens*		O-220306 (H136)	KY772987	KY784269	KY772877
*H. pallescens*		O-289039 (H070)	KY772953	KY784232	KY772862
*H. panormitana*	Epitype	O 253363 (H064)	KY772948	KY784228	KY772856
*H. panormitana*		O-65394 (H143)	KY772992	KY784276	/
*H. panormitana*		O-203499 (H145)	KY772994	KY784278	/
*H. rugosa*		Zhao 8021	MG847045	/	MG847091
*H. rugosa*		HKAS 87587	MG871320	/	MG980690
*H. rugosa*		Zhao 482	KT932631	/	/
*H. rugosa*		MFLU23-0094	OQ866051	OQ863533	OQ863549
*H. rugosa*		MFLU23-0095	OQ866052	OQ863534	/
*H. rugosa*		MFLU23-0093	OQ866050	OQ863532	OQ863548
*H. sublactea*	Holotype	zhao1032	KT894832	/	/
*H. sublactea*		zhao1273	KT894834	/	/
*H. subspadicea*	Holotype	HKAS 56656	NG_059663	/	KU739848
*H. subspadicea*		HKAS 90624	KU739822	/	KU739849

The newly generated sequences are indicated with red text. The symbol “/” denotes no available data.

A maximum likelihood (ML) analysis was performed using IQ-Tree (http://iqtree.cibiv.univie.ac.at/) (Trifinopoulos et al., 2016). The substitution model options for each gene were auto-evaluated according to the provided partition file. Clade support for the ML analysis was assessed using an SH-aLRT test with 1,000 replicates (Guindon et al., 2010) and the ultrafast bootstrap (UFB) (Hoang et al., 2018). In the ML analysis, nodes with support values of both SH-aLRT ≥ 80 and UFB ≥ 95 were considered well-supported, those with either SH-aLRT < 80 or UFB < 95 were weakly supported, and nodes with both SH-aLRT < 80 and UFB < 95 were unsupported.

Bayesian inference (BI) (Rannala and Yang, 1996) was performed with Markov Chain Monte Carlo sampling (MCMC) to evaluate the posterior probability using MrBayes on XSEDE (3.2.7a) with default parameters on CIPRES Science Gateway (https://www.phylo.org/). The number of generations was set at 5,000,000, sampling every 1,000 generations and a burn-in value of 25%. Nodes were considered strongly supported with posterior probability values >0.90. The resulting phylogenetic tree was visualized in Figtree v.1.4.4 program (Rambaut, 2018).

## 3. Results

### 3.1. Phylogenetic analyses

Phylogenetic analyses were based on a combined dataset of three-gene sequences from 55 taxa representing 19 species in *Helvella s.s*. Two species in *Dissingia* were outgroups. The combined dataset comprises 1,992 characters with gaps (LSU: 1-679, *HSP*90: 680-913, *TEF*: 914-1,429), of which 1,105 characters were constant, 287 characters were parsimony-informative, and 37 were singleton sites. The estimated base frequencies were as follows: A = 0.245313, C = 0.259607, G = 0.279860, T = 0.215221, substitution rates AC = 1.014740, AG = 3.751983, AT = 1.042703, CG = 1.094652, CT = 9.693442, and GT = 1.000000; and gamma distribution shape parameter α = 0.180781. The ML and BI analyses resulted in phylogenetic trees with a similar topology. Thus, the topology from the ML tree is presented along with statistical values from the SH-aLRT/UFB/BIPP algorithms.

**Figure 1:**
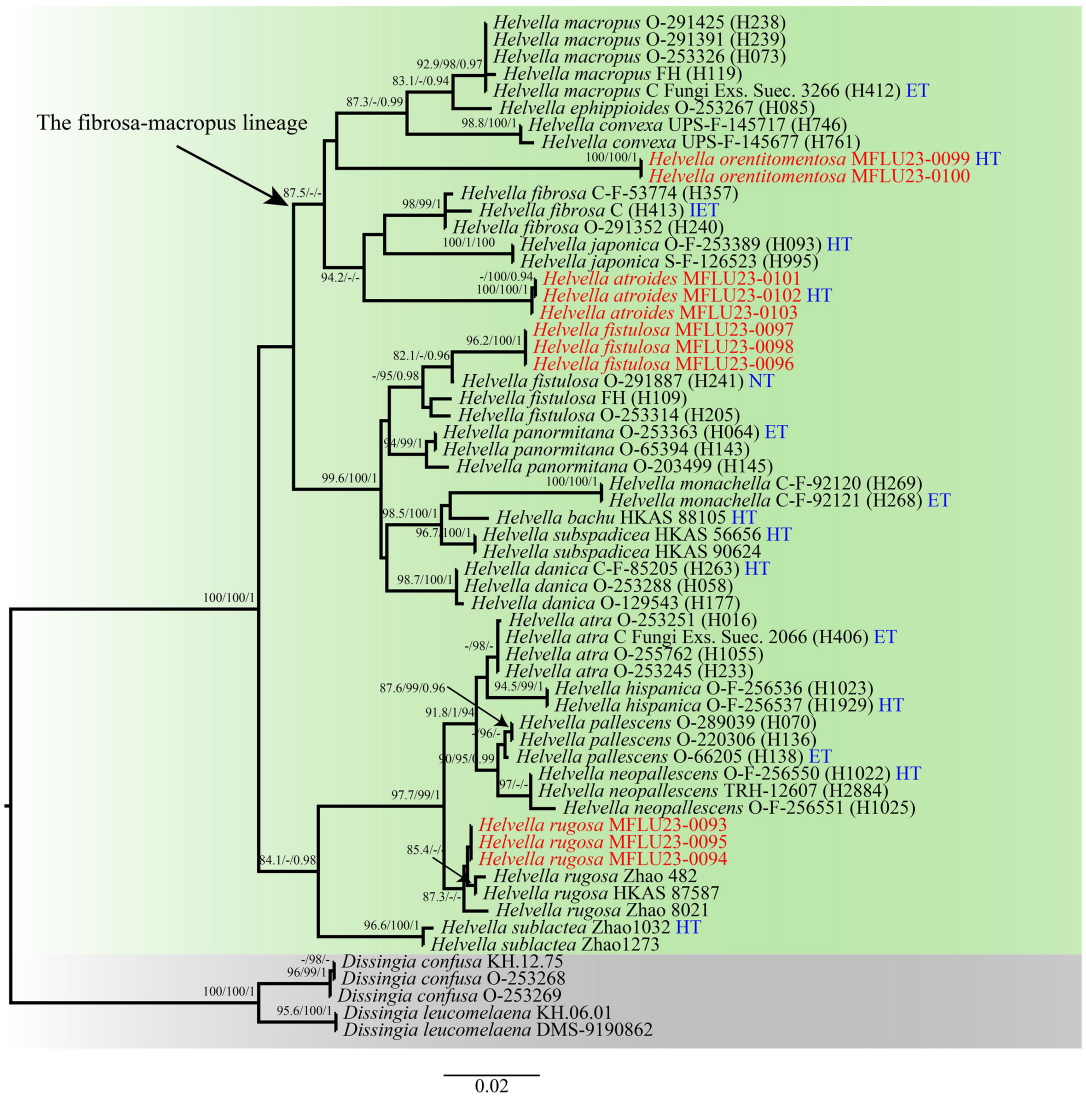
Maximum likelihood (ML) tree of *Helvella* and its allies within *Helvellaceae* inferred from combined LSU, *TEF*, and *HSP*90 datasets. Bootstrap support values for ML ≥ 80 of SH-aLRT or 95 of UFB and posterior probability for BI ≥ 0.90 are indicated above the nodes and separated by ‘-/-/-' (SH-aLRT/UFB/BIPP). Specimens of the current study are given in red. Type specimens are in bold. The letter ET stands for epitype, HT for holotype, IET for isoepitype, and NT for neotype.

The phylogeny shows that our newly collected samples formed four evolutionary lineages (Figure 1). Two independent clades were discerned as new species to science, i.e., *H. atroides* and *H. orentitomentosa*. They are nested in the fibrosa-macropus lineage, which now consists of seven species. This lineage exhibits a broad spectrum of apothecial shapes, from regularly cupulate to saddle-shaped to lobed capitate. *Helvella macropus, H. ephippioides, H. convexa*, and *H. orentitomentosa* constitute one lineage and *H. fibrosa, H. japonica*, and *H. atroides* constitute a sister lineage. Samples of *H. orentitomentosa* (Thailand) were inferred as a monophyletic clade, which is a successive sister group to *H*. *convexa* (Finland, Sweden), *H. ephippioides* (Japan, Sweden), and *H. macropus* [Asia (China), Europe, and North America]. *Helvella atroides* formed an independent lineage separated from *H. japonica* (Japan, Norway, and Sweden) and *H. fibrosa* (Asia and Europe). In addition, two geographic distributions of *H. fistulosa* and *H. rugosa* are new records in Thailand. Three samples (MFLU23-0093, MFLU23-0094, and MFLU23-0095) were clustered with *H. rugosa*, indicating that these taxa were homogeneous with *H. rugosa*. The newly collected samples MFLU23-0096, MFLU23-0097, and MFLU23-0098 were nested in the widely distributed *H. fistulosa*.

### 3.2. Taxonomy

**Figure 2:**
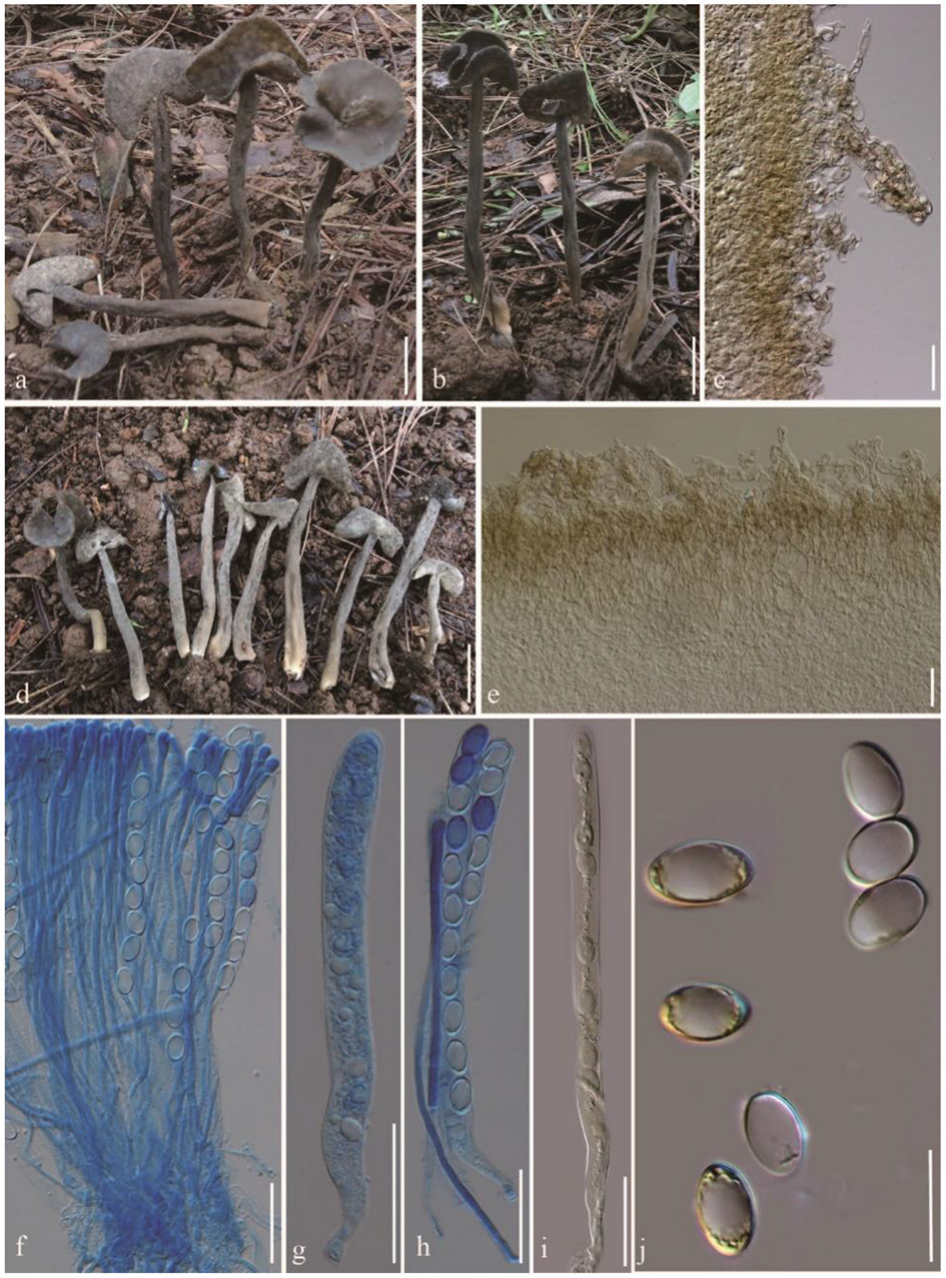
*Helvella atroides*. **(a, b, d)** Typical mature specimens [**(a, b)** MFLU23-0103, **(d)** MFLU23-0101]; **(c)** receptacle surface of pileus; **(e)** stipitipellis; **(f)** asci and paraphyses; **(g–i)** asci; **(j)** ascospores. Scale bars. **(a, b, d)** = 1 cm; **(c, e–i)** = 50 μm; **(j)** = 20 μm.

***Helvella atroides*** Q. Zhao, sp. nov. (Figure 2).

Index Fungorum number: IF 551934, Facesoffungi number: FoF 01337.

*Etymology*: *atroides* named as its gross morphology is similar to *H. atra*.

*Diagnosis*: Saddle shaped, slightly acetabuliform, irregularly lobed to discoid pileus, margin upward, glabrous hymenium dark gray to brownish black, villose receptacle surface gray to dark gray, terete stipe solid, with one or more longitudinal grooves, pubescent to villose, concolorous with receptacle surface. Asci 200–270 × 13–15 μm, pleurorhynchous. Paraphyses 5–6 μm wide at the apex. Ascospores 17–20.5 × 10–12.5 μm, ellipsoid.

*Typification*: THAILAND, Chiang Rai Province, Mai Sai District, on the ground under *Pinus kesiyi* Royle ex Gordon, alt. 640 m, 31 Aug 2015, Q. Zhao, Zhao 2672 (MFLU23-0102: **holotype**).

Apothecia saddle-shaped to slightly acetabuliform when young, irregularly lobed in age, up to 2 cm high, 1–2 cm broad, margin upward; hymenium even, dark gray to brownish black, becoming black when dried; receptacle surface villose to tomentose, gray to dark gray, sometimes possessing a few mottled gray pigments, becoming grayish when dried. Stipe 4–8 cm long, 0.4–0.6 cm thick, terete, solid, with one or more longitudinal grooves, pubescent to villose, concolorous with receptacle surface, paler to yellowish near the base. Medullary excipulum 100–150 μm broad, hyaline, composed of 3.5–5 μm broad hyphae, J^−^. Ectal excipulum 50–150 μm broad, outermost cells catenuliform in long fascicled tufts, hyaline, evenly blue in cotton blue, with cylindrical to subclavate, slightly thick-walled end cells 15–35 × 9–13 μm, J^+^. Stipitipellis 180–350 μm, hyaline, terminal cells 15–50 × 8–14 μm, clavate, J^+^. Asci 200–280 × 13–16 μm, pleurorhynchous, 8-spored, subcylindrical to clavate. Paraphyses filiform, 4–5 μm broad, slightly exceeding the asci, apex obviously enlarged, 6–8 μm broad, deeply blue in cotton blue, J^−^. Ascospores [60/3/3, in H_2_O] (16–) 17–20.5 (−21) × 10–12.5 (−13) μm, Q = (1.5–) 1.32–1.8, **Q** = 1.64 ± 0.08, ellipsoid, smooth-walled under the light microscope.

*Habitat*: Solitary, scattered, or gregarious on the ground under *Pinus kexiya*.

*Additional specimens examined*: Thailand. Chiang Rai Province, Mai Sai District, alt. 640 m, 31 Aug 2015, Q. Zhao, Zhao 2670 (MFLU23-0103), Zhao 2676 (MFLU23-0101).

*Notes*: In phylogenetic analysis (Figure 1), *H. atroides* formed an independent branch and grouped as a sister clade to *H. japonica* and *H. fibrosa*. Morphologically, *H. atroides* can be distinguished from *H. japonica* by its mottled gray pigments of apothecia and from *H. fibrosa* by its darker hymenium and stipe color. Microscopically, spores of *H. fibrosa* (14.3–16.2 μm) are shorter than that of *H. atroides* (17–20.5 μm). Moreover, they can also be separated by the size of paraphyses, with paraphyses of *H. fibrosa* being slimmer than that of *H. atroides* (Kaygusuz et al., 2020).

The gross morphology of *H. atroides* is very similar to that of typical *H. atra* J. König *s.s*. However, the hymenial surface and stipe of *H. atroides* are usually dark gray to brownish black when young, while in the latter species, it is generally completely black (Landeros et al., 2012).

**Figure 3:**
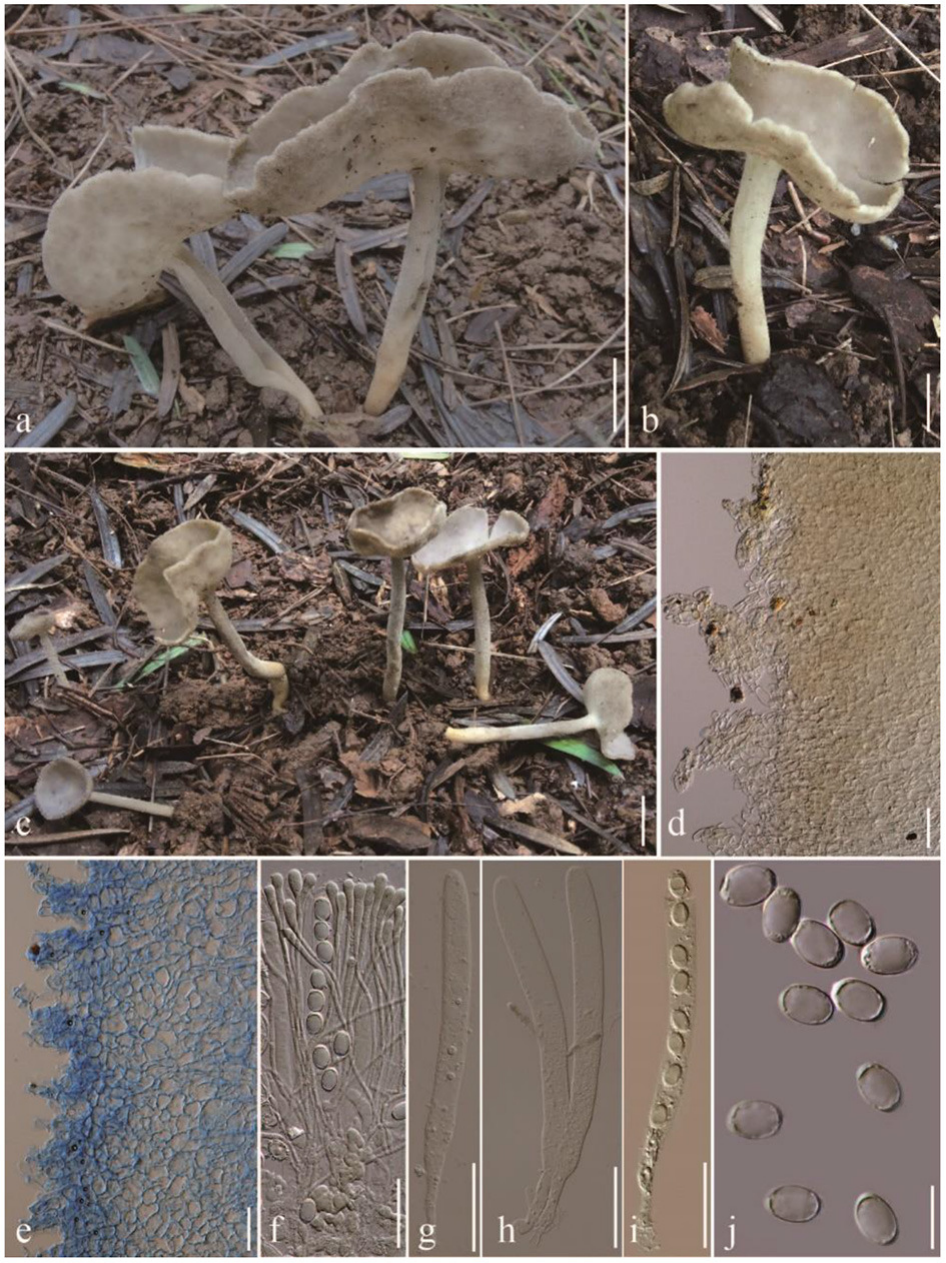
*Helvella orentitomentosa*. **(a–c)** Typical mature specimens [**(a, b)** MFLU23-0099, **(c)** MFLU23-0100]; **(d)** receptacle surface of pileus; **(e)** stipitipellis; **(f)** asci and paraphyses; **(g–i)** asci; **(j)** ascospores. Scale bars. **(a–c)** = 1 cm; **(d, e)** = 50 μm; **(f–i)** = 20 μm.

***Helvella orentitomentosa*** Q. Zhao **sp. nov**. (Figure 3).

Index Fungorum number: IF 551937, Facesoffungi number: FoF 01340.

*Etymology*: *orentitomentosa* refers as its tomentose receptacle surface and stipe surface in oriental region.

*Diagnosis*: Pileus cupulate to lightly cupulate when young, irregularly lobed to discoid in age, even hymenium gray, tomentose receptacle surface gray, tomentose stipe terete, solid, gray. Asci 260–360 × 14–18 μm, ellipsoidal ascospores 16–19 × 11–13 μm, obviously enlarged paraphyses apex 8–11.5 μm broad.

*Typification*: Thailand. Chiang Rai Province, Mai Sai District, in conifer forest dominated by *Pinus kesiyi*, alt. 640 m, 31 Aug 2015, Q. Zhao, Zhao 2668 (MFLU23-0099: **holotype**).

Apothecia cupulate to lightly cupulate when young, irregularly lobed to discoid when mature, up to 5 cm high, 1–3 cm broad; margin slightly undulate; hymenium even, gray, becoming grayish when dried; receptacle surface tomentose, without ribs, gray, becoming grayish when dried. Stipe 4–6 cm long, 0.4–0.7 cm thick, terete, tomentose, above concolorous with receptacle surface, below gradually becoming paler to yellowish white near the base; basal mycelium white. Medullary excipulum 180–300 μm broad, hyaline, composed of 7–10 μm broad hyphae, J^−^. Ectal excipulum 70–200 μm broad, outermost cells catenuliform in long fascicled tufts, hyaline, evenly blue in cotton blue, with cylindrical to subclavate, slightly thick-walled end cells 20–55 × 12–30 μm, J^−^. Stipitipellis 200–320 μm, hyaline, terminal cells 18–31 × 8–15 μm, clavate, J^−^. Asci 210–250 × 16–18 μm, pleurorhynchous, 8-spored, subcylindrical to clavate. Paraphyses filiform, 3–4.5 μm broad, slightly exceeding the asci, apex obviously enlarged, 8–11.5 μm broad, blue in cotton blue, J^−^. Ascospores [60/4/4, in H_2_O] (15–) 16–19 (−20) × 11–13 (−14) μm, Q = (1.23–) 1.37–1.67, **Q** = 1.50 ± 0.06, ellipsoid, smooth-walled under the light microscope.

*Habitat*: Solitary, scattered, or gregarious on the ground, and in conifer forest dominated by *Pinus* sp.

*Additional specimens examined*: Thailand. Chiang Rai Province, Mai Sai District, in conifer forest dominated by *Pinus kesiyi*, alt. 640 m, 31 Aug 2015, Q. Zhao, Zhao 2669 (MFLU23-0100).

*Notes*: In phylogeny (Figure 1), *H. orentitomentosa* is close to *H. convexa, H. ephippioides*, and *H. macropus*. In morphology, the hymenium of *H. orentitomentosa* is gray, that of *H. convexa* is brownish black to black, that of *H. ephippioides* is gray, smoky, hazel, or sooty, rarely whitish (Imai, 1932), and that of *H. macropus* is yellowish to grayish brown (Skrede et al., 2017). Microscopically, compared with *H. ephippioiDes* (250–322 × 15–19 μm, 20–25 × 10 μm), *H. orentitomentosa* has shorter asci, as well as the shorter and wider ascospores. Comparing with *H. orentitomentosa, H. macropus* (240–300 × 13–16 μm) and *H. convexa* (260–295 × 10.2–13.0 μm) have longer and slender asci, *H. macropus* has longer ascospores (19.5–23.4–25.8 in length), and *H. convexa* has narrower ascospores (8.4–9.0–9.6 μm in width). The ascospores of *H. orentitomentosa* and *H. convexa* are ellipsoid, while those of the other two species are ellipsoid-fusoid or ellipsoid-subfusiform. When old, *H. orentitomentosa* sometimes can be indistinguishable from *H. atroides*. However, they have a different medullary excipulum thickness, which is 180–320 μm in *H. orentitomentosa* and 100–150 μm in *H. atroides* (Figure 3).

### 3.3. New geographic distribution records

**Figure 4:**
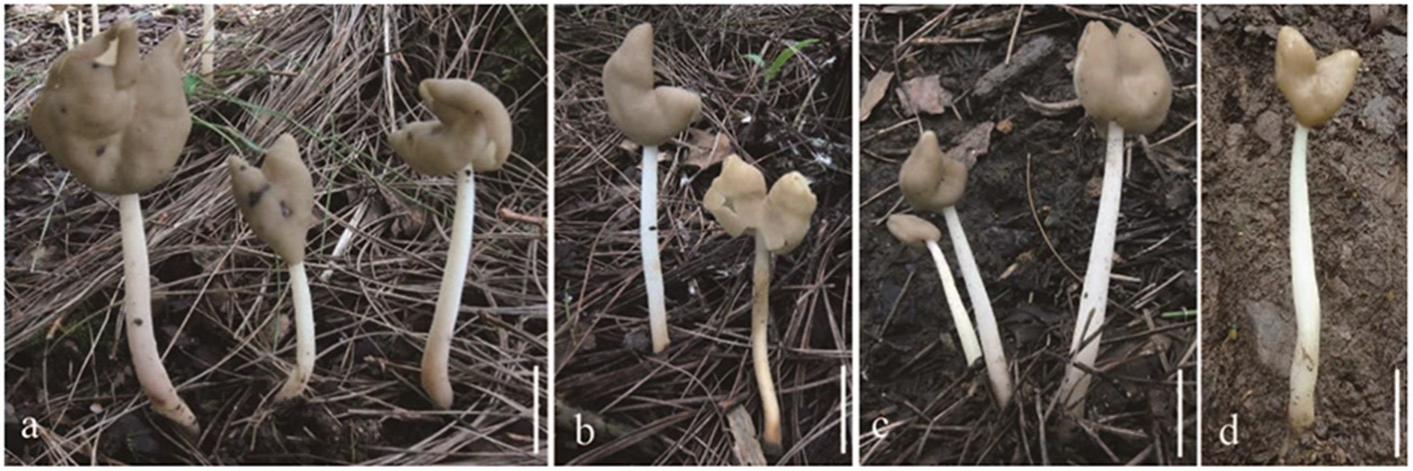
*Helvella fistulosa*. **(a–d)** Typical mature specimens [**(a, b)** MFLU23-0097, **(c)** MFLU23-0098, and **(d)** MFLU23-0096]. Scale bars. **(a–d)** = 1 cm.

***Helvella fistulosa*** Alb. & Schwein., Consp. Fungorum Lusat. 299 (1805) (Figure 4).

Index Fungorum number: IF 204780, Facesoffungi number: FoF 01339.

Apothecia saddle-shaped, 0.5–3 cm high, 0.7–3 cm broad, margins sometimes fused, but always free from the stipe; hymenium glabrous, cream to taupe, becoming yellowish when dried; receptacle surface finely undulate, white to cream, yellowish when dried. Stipe 6–11 cm long, 0.3–0.6 cm broad, terete, more or less equal, solid to hollow, finely pubescent, white to cream, yellowish when dried, internally white. Medullary excipulum 180–270 μm broad, hyaline, composed of thick-walled 4–5 μm broad hyphae, J^−^. Ectal excipulum 90–140 μm broad, outermost cells catenuliform in long fascicled tufts, hyaline, evenly blue in cotton blue with cylindrical to subclavate, end cells 22–40 × 10–22 μm, slightly thick-walled, J^−^. Stipitipellis 80–120 μm, hyaline, terminal cells 10–30 × 8–12 μm, subglobose to subclavate, J^−^. Asci 270–310 × 15–18 μm, pleurorhynchous, 8-spored, subcylindrical to clavate. Paraphyses filiform, 3–4 μm broad, slightly exceeding the asci, apex slightly enlarged, 4–7 μm broad, blue in cotton blue, J^−^. Ascospores [60/3/3 in H_2_O] 19–23 (−24) × (11–)12–14(−15) μm, Q = (1.33–) 1.38–1.73 (−1.77), **Q** = 1.53 ± 0.09, ellipsoid, smooth-walled under the light microscope.

*Habitat:* Solitary, scattered, or gregarious on the ground under *Pinus armandii*.

*Specimens examined*: Thailand. Chiang Rai Province, Mai Sai District, alt. 640 m, 31 Aug 2015, Q. Zhao, Zhao 2671 (MFLU23-0098), Zhao 2675 (MFLU23-0096), Zhao 2673 (MFLU23-0097).

*Notes*: *Helvella fistulosa* was first reported in Thailand. Skrede et al. (2017) checked some specimen named *Helvella fistulosa* from Iceland, Japan, Norway, and the United States. They re-described *H. fistulosa* and designated a Norwegian specimen labeled O-291887 (H241) as the neotype. In this study, three newly added collections from Thailand were clustered together and nested within *H. fistulosa* taxa with a strong support value in our phylogeny. Compared with the neotype of *H. fistulosa*, the hymenium of our samples has a lighter color (Skrede et al., 2017). There is no significant morphological difference between our samples and the neotype in microscopic characteristics.

**Figure 5:**
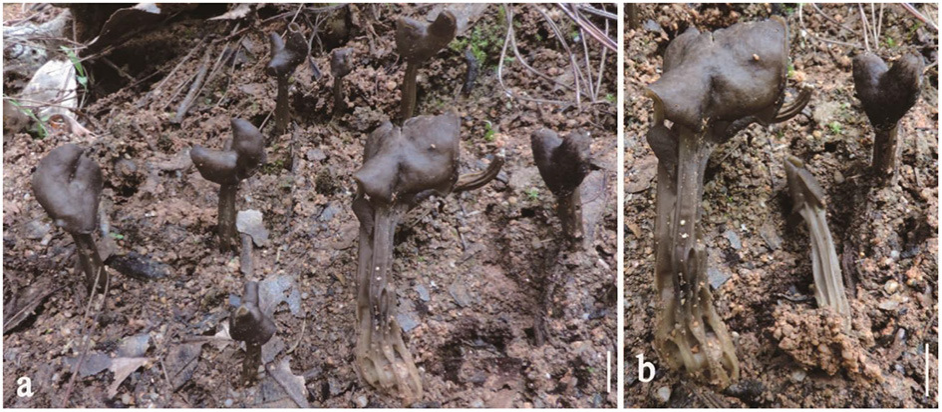
*Helvella rugosa*. **(a, b)** Typical mature specimens [**(a, b)** Zhao 2664 MFLU23-0093]. Scale bars. **(a, b)** = 1 cm.

***Helvella rugosa*** Q. Zhao & K. D. Hyde, Fungal Diversity 75: 142 (2015) (Figure 5).

Index Fungorum number: IF 551447, Facesoffungi number: FoF 00972.

Apothecia saddle-shaped to three-lobed, 1–2 cm high, 1–2 cm broad, margin reflexed and fused with the stipe, hymenium glabrous, light brown to dark gray or blackish brown when fresh, becoming black when dried, receptacle surface wrinkled-folded, white to pale to smoky when young, becoming yellowish when dried. Stipe 2–7 cm long, 0.4–0.8 cm broad, tapering downwards, lacunose, with sharped ribs, glabrous, grayish brown when young, becoming black when dried. Medullary excipulum 80–250 μm broad, hyaline, hyphae 3–5 μm broad, enlarged cells 8–15 × 7–14 μm, walls thickened, J^−^. Ectal excipulum 50–80 μm broad, outermost cells paliform, hyaline, terminal cells 16–40 × 8–16 μm, J^−^. Stipitipellis 50–70 μm, hyaline, terminal cells 18–32 × 8–18 μm, walls thickened, J^−^. Asci 220–260 × 13–17 μm, pleurorhynchous, 8-spored, subcylindrical to clavate. Paraphyses filiform, 4–5 μm broad, slightly exceeding the asci, apex enlarged, 6–8 μm broad, brown, J^−^. Ascospores [80/4/4, in H_2_O] 15–18.5 × 10–12 (−12.5) μm, Q = (1.36–) 1.4–1.71 (−1.75), **Q** = 1.51 ± 0.06, ellipsoid, smooth-walled under the light microscope.

*Habitat*: Scattered or gregarious on the ground, and in deciduous forests of *Quercus* sp.

*Specimens examined*: Thailand. Chiang Mai Province, Mushroom Research Center, in deciduous forest dominated by *Quercus* spp., alt. 740 m, 22 Aug 2015, Q. Zhao, Zhao 2660 (MFLU23-0094), Zhao 2662 (MFLU23-0095), Zhao 2664 (MFLU23-0093).

*Notes*: *Helvella rugosa*, usually found in deciduous forests, was known only from China (Ariyawansa et al., 2015). Our samples extend their distribution to Thailand. All samples from Thailand are similar to the original description (Ariyawansa et al., 2015), and their ecological preference is associated with the coniferous forest dominated by *Quercus* spp.

## 4. Discussion

Species delimitation, taxonomy, and typification in saddle-like fungi have always been challenging. Traditionally, the macroscopic characteristics of the hymenium, receptacle surface appendage, excipulum, and stipe have been used as the key diagnostic characters to distinguish *Helvella* from its allied species (Dissing, 1966; Korf, 1972; Weber, 1972; Harmaja, 1979; Häffner, 1987; Abbott and Currah, 1997; Nguyen et al., 2013). In Dissing's research, the importance of paraphysis pigmentation was emphasized, which was reflected in the color of hymenium. However, the hymenium color varies greatly in some species, from pale grayish brown to brown to almost black, e.g., *H. elastica, H. lacunosa*, and *H. rugosa* (Dissing, 1966; Ariyawansa et al., 2015). Skrede et al. (2017) indicated that the color of fresh apothecia could change dramatically during drying. In microanatomical features, the characteristics of ectal excipulum, especially the shape, color, and distribution of the outer hyphoid hairs, are of special diagnostic value in morphologically similar species (Landeros et al., 2012). In addition, the ascus base (aporhynchous or pleurorhynchous), as well as the pigmentation and the shape of paraphyses upperpart also helped in species discrimination of some Helvella (Landeros et al., 2012; Skrede et al., 2017).

The development of molecular systematics and the possibility of employing DNA barcode sequences as a more robust tool to identify specimens of closely related species have been applied to taxonomic studies of *Helvella* (Skrede et al., 2017). As a universal barcode for fungi identification, ITS is not suitable for addressing the phylogeny and species discrimination across the *Helvella*, because their ITS regions (especially ITS1) are too divergent to be arranged within the whole genus, as well as the limited molecular information of ITS fragments in GenBank (Landvik et al., 1999; Wang et al., 2019). Skrede et al. (2017) showed that genetic markers LSU, *RPB*2, and *HSP*90 provided useful barcodes for species delimitation in *Helvella*, due to their moderate sequence length, high amplification success rate, and reasonable phylogenetic informative properties. Later, Wang et al. (2019) focused on the rib-stiped cupulate species of *Helvella* and revealed that the success rate of using primers ITS3/ITS4 for ITS2 region amplification was much higher than that of using primers ITS5/ITS4 for ITS gene amplification. The authors proposed that *HSP*90 and ITS2 should be used as supplementary DNA barcodes for the cupulate *Helvella* species with ribbed stipe (Wang et al., 2019). With the help of molecular systematics, in the past decade, some researchers have discriminated some novel species and re-evaluated the circumscription of morphologically similar species or pseudo-cryptic species or complex groups (Nguyen et al., 2013; Ariyawansa et al., 2015; Landeros et al., 2015; Zhao et al., 2015, 2016a,b; Skrede et al., 2017, 2020; Hansen et al., 2019; Xu et al., 2022).

Ecologically, hosts are also important traits for the taxonomy and phylogeny of ectomycorrhizal and have been used to recognize the species of *H. dryophila, H. pseudolacunosa, H*. *rugosa*, and *H. vespertina* (Nguyen et al., 2013; Ariyawansa et al., 2015). *Helvella lacunosa* in China is mainly associated with *Betula* sp., *Dryas* sp. and *Salix* sp., and those specimens collected from Sweden are mainly associated with *Betula* sp., *Dryas* sp., *Fagus* sp., *Quercus* sp., and *Malus* sp. (Dissing, 1966). Skrede et al. (2017) found that *H. arctoalpina* and *H. dryadophila* are always closely associated with *Dryas* species while *H. fusca* with *Populus* species. Our newly described species, *H. atroides* and *H. orentitomentosa*, may have mycorrhizal host specificity because they are all found in coniferous forest, such as *Pinus* spp. However, whether these ectomycorrhizal features can be reflected in the morphology and systematics of saddle fungi remains to be determined.

In this study, based on analyses of combined LSU, *HSP*90, and *TEF* sequence data, two new species and two new geolocation records in Thailand were identified and described. In the phylogenetic tree (in Figure 1), the newly collected samples from Thailand are scattered among the taxa that are distributed in other continents, mostly from Europe. Previous research also revealed that *Helvella* samples from different regions encompass many morphologically similar but distinct phylogenetic species (Skrede et al., 2017). In *Helvella*, some endemic species are only distributed in a narrow area (e.g., *H*. *bachu, H. subspadicea*, and *H*. *zhongtiaoensis*), while others can be widespread across the world (e.g., *H. alpestris, H. capucina, H. solitaria, H. phlebopora*, and *H. fistulosa*) (Zhao et al., 2015, 2016a; Skrede et al., 2017). In tropical Thailand, together with the four newly added species in this study, a total of seven saddle fungi are distributed here. A key to Thai saddle fungi is given.

### 4.1. Key to the species of *Helvella* known from Thailand

1a Stipe terete, sometimes with slight folds, but never with true ribs………………… 2
1b Stipe ribbed, deeply ribbed or lacunose .…………… 5
2a Pileus saddle-shaped to irregularly-lobed, but never cupulate……… 3
2b Pileus cup-shaped or lobed……………………. . 4
3a Stipe whitish to yellowish; asci 330–360 × 15–17 μm ………… *H. elastica*
3b Stipe yellowish, and occasionally reddish brown when dried; asci 270–310 × 17–18 μm .……………… *H. fistulosa*
4a Hymenium grey, receptacle surface greyish; stipe grey, becoming greyish when dried; asci 210–250 × 16–18 μm……………… *H. orentitomentosa*
4b Hymenium blackish, receptacle surface grey brown to pale brown; stipe grey to dark grey, paler to yellowish near the base; asci 200–280 × 13–16 μm…………. . . *H. atroides*
5a Hymenium cream to brownish-yellow, receptacle surface creamy white, light greyish to brownish orange; stipe white, cream to pale yellowish……………………… . . 6
5b Hymenium light brown to dark gray or blackish brown to black, receptacle surface white, pale, and smoky to yellowish; stipe glabrous, grayish brown to black………. . *H. rugosa*
6a Ascospores 18–21 × 11–12.5 μm…………. . H. crispa
6b Ascospores 15–17 × 9.5–11.5 μm .………. . *H. crispoides*

## Data availability statement

The datasets presented in this study can be found in online repositories. The names of the repository/repositories and accession number(s) can be found in the article/supplementary material.

## Author contributions

F-MY, LL, TL, QZ, and Y-AZ: design of the research, writing and revising the manuscript, data analysis, and interpretation. F-MY and QZ: performance of the research. QZ: collection of materials. All authors contributed to the article and approved the submitted version.
